# Minimally invasive plate osteosynthesis (MIPO) versus open reduction and internal fixation (ORIF) in the treatment of distal fibula Danis-Weber types B and C fractures

**DOI:** 10.1186/s13018-020-02018-5

**Published:** 2020-10-22

**Authors:** Cesare Marazzi, Matthias Wittauer, Michael T. Hirschmann, Enrique A. Testa

**Affiliations:** 1 Department of General Surgery, Hospital Oberengadin, Samedan, Switzerland; 2 University of Basel, Basel, Switzerland; 3 Department of Orthopaedic and Trauma Surgery, University Hospital Basel, Basel, Switzerland; 4 Department of Orthopaedic Surgery and Traumatology, Kantonsspital Baselland (Bruderholz, Liestal, Laufen), Bruderholz, Switzerland; 5 Department of Orthopaedic and Trauma Surgery, Hospital Lugano, Lugano, Switzerland

**Keywords:** Ankle fracture, Fibula fracture, Minimally invasive, MIPO, Open reduction, ORIF, Complications

## Abstract

**Background:**

Minimally invasive plate osteosynthesis (MIPO) has been reported to be superior to open reduction and internal fixation (ORIF) in the treatment of different long bone fractures. Nevertheless, in distal fibula fractures, the evidence of MIPO remains scarce. The aim of this retrospective study was to compare the clinical and radiological outcomes of the minimally invasive techniques applied to the distal fibula with open reduction and internal fixation within a 12 months follow-up.

**Methods:**

A consecutive series of patients who underwent surgery using either ORIF or MIPO for the treatment of distal fibula fractures between 2010 and 2014 were retrospectively analyzed. All distal fibular fractures requiring an operative treatment (Danis-Weber type B ≙ AO type 44 B1, 2, 3 and Danis-Weber type C ≙ AO type 44 C1, 2) were included (ORIF *n* = 35, MIPO *n* = 35). Patients were assessed for postoperative pain using a visual analog scale (VAS) for pain (ranging from 0 to 10) and classified into 4 groups: “no pain” for VAS = 0, “low” for VAS = 1–3, “moderate” for VAS = 3–5, and “severe” for VAS = 5–10. In addition, complications of postoperative fracture-related infection, wound healing disorders, vascular and nerve injury and development of nonunion were evaluated and analyzed. Radiologic outcome measures assessing the talocrural angle, lateral and medial clear space, tibiofibular overlap, and talar tilt angle were evaluated postoperatively.

**Results:**

The overall complication rate showed to be lower in the MIPO group compared to the ORIF group (14% vs. 37%, *p* = 0.029). Even though not statistically significant, specific surgery-related complications such as skin necrosis (3% vs. 9%, *p* = 0.275), nonunion (0% vs. 6%, *p* = 0.139), infections and wound healing disorders (9% vs. 20%, *p* = 0.141), as well as postoperative pain (17% vs. 26%, *p* = 0.5) were found more frequently in the ORIF group. The tibiofibular overlap demonstrated to be significantly lower in the ORIF group (3.3 mm vs. 2.7 mm, *p* = 0.033). The talocrural angle, talar tilt angle, and lateral and medial clear space showed to be equivalent in both groups.

**Conclusion:**

In this retrospective single-center consecutive series, MIPO was superior to ORIF in the surgical treatment of distal fibula fractures with respect to the overall complication rate.

**Trial registration:**

EKNZ Project-ID: 2019-02310, registered on the 20th of December 2019 with swissethics

## Background

Ankle injuries are among the most common traumatic pathologies treated in emergency departments worldwide, and lateral malleolus fractures represent one of the most common indications for open reduction and internal fixation (ORIF) [1–3].

The ORIF of complex ankle fractures is a demanding procedure and associated with a considerable number of complications, mainly because of the thin soft tissue and skin layer covering the bone in this particular region [4–7].

The importance of respecting the biological status in the management of fractures is nowadays emphasized by a large number of publications investigating the possible harm of standard approaches on surrounding soft tissues [3, 7–11].

In the last decades, MIPO (minimally invasive plate osteosynthesis) techniques became widely successful with the launch of angular stable screw-plate systems like LISS (less invasive stabilization system) or LCP (locking compression plate), mainly to treat long bone fractures minimally invasively. Despite some drawbacks, such as the impossibility to manipulate and assess the fracture site under direct vision [12], current medical literature suggests that closed reduction and submuscular plating techniques through percutaneous insertion have comparable fracture healing rates and less percentages of non-union than ORIF [3, 13–15].

Despite the increasing number of publications in the last 10 years investigating MIPO for femoral and tibial fractures, to date there is only a paucity of studies dealing with the treatment of distal fibula fractures using MIPO technique [2–4, 8, 16–18]. Of those studies, there is only one that directly compares MIPO to ORIF [4]. Nevertheless, those studies could show a decreased risk for nonunion and infection when minimally invasive techniques where applied to treat distal fibula fractures.

This retrospective study compares the clinical and radiological outcome and complications after MIPO to the traditional open reduction and internal fixation in the treatment of Danis-Weber types B and C fractures of the distal fibula (AO Type 44 B 1-2-3 and 44 C 1-2).

The hypothesis was that superior results might be achieved with MIPO regarding clinical and radiological outcomes as well as lower complication rates in comparison with ORIF. The purpose of this study was to create more evidence answering the question which operative technique is best in the largest retrospective cohort study.

## Methods

This retrospective study was performed in a regional trauma hospital in Delémont, Switzerland. The study was approved by the local ethical committee and conducted following the STROBE guidelines [19]. Patients who underwent surgery using ORIF or MIPO for the treatment of distal fibula fractures between 2010 and 2014 were consecutively included. These patients were then retrospectively divided into two groups according to the operative technique used.

Fractures were classified using the Danis-Weber classification as recommended by both the Orthopaedic Trauma Association (OTA) and the Arbeitsgemeinschaft für Osteosynthesefragen (AO Foundation) [20, 21].

Inclusion criteria were all distal fibular fractures requiring an operative treatment (Danis-Weber type B ≙ AO type 44 B1, 2, 3 and Danis-Weber type C ≙ AO type 44 C1, 2). Exclusion criteria were complex pilon fractures (AO43C3), Maisonneuve fractures (AO44C3), bilateral leg fractures, and patients who had undergone previous surgery at the fracture site. In addition, patients were excluded if they suffered from existing disorders that might affect healing process and function, such as congenital deformities or neurologic disorders.

In total, 70 patients matched the inclusion criteria and were finally included in the present study. The baseline demographic and clinical data are shown in Table 1. All surgeries were done by one of the four senior surgeons of the institution, all of them with minimum 10 years of experience in trauma surgery.

**Table 1:** Demographic and clinical data at baseline

Factor	MIPO (***n*** = 35)	ORIF (***n*** = 35)	***t*** test	***p*** value
Age at injury (years)	54.8 ± 17.6	52.2 ± 11.3	*t* = 0.731	0.467
Male sex (*n*)	17 (49%)	12 (34%)	*I*^2^ = 1.472	0.225
Osteoporosis (*n*)	1 (3%)	2 (6%)	*I*^2^ = 0.348	0.555
Peripheral artery disease (*n*)	1 (3%)	1 (3%)	*I*^2^ = 0.000	1.000
Diabetes mellitus (*n*)	3 (9%)	2 (6%)	*I*^2^ = 0.215	0.643
Smoking (*n*)	6 (17%)	8 (23%)	*I*^2^ = 0.357	0.550
Fracture classification (*n*)	Weber B = 29 Weber C = 6	Weber B = 29 Weber C = 6	*I*^2^ = 0.000	1.000
Level of energy of trauma (*n*)	High = 8 (23%) Medium = 4 (6%) Low = 25 (71%)	High = 4 (11%) Medium = 4 (11%) Low = 27 (77%)	*I*^2^ = 2.077	0.354
Ex-fix placement (*n*)	9 (26%)	11 (31%)	*I*^2^ = 0.280	0.597

All variables were reported in terms of counted cases and relevant percentages and compared with the *I*^2^ test, with the exception of age at trauma reported in terms of mean and standard deviations and compared by means of *t* test

For soft tissue evaluation, an empiric classification was used, correlating the local status with the energy of trauma and creating three different groups. “Low” for minor trauma with mild soft tissue lesion, “medium” for pro-supination trauma or direct contusion trauma with no open fracture associated, and “high energy trauma” for motor vehicle collisions with massive soft tissue involvement or open fractures.

### Description of surgical technique

For both, open reduction and internal fixation (ORIF) and minimally invasive plate osteosynthesis (MIPO), the surgical recommendations of the AO Foundation were strictly followed [22].

Surgery was done after soft tissue swelling had settled. In case of high-energy trauma or subluxated fracture with prominent swelling, a two-step approach, using a temporary fracture stabilization with an external fixator, was performed.

Patients of both groups were placed in supine position (supine with a bump under the ipsilateral hip with the knee slightly flexed) on a radiolucent table. If an external fixator was in place, all the bars and pins were removed.

In the ORIF group, an open surgical approach was established. The skin incision was lateral to the fibula and slightly anterior if additional access to the anterior syndesmosis was required. The area of the fracture was uncovered and gently reduced with one or two Weber clamps. If required, a lag screw was inserted. Then, a plate was placed according to the AO technique. Depending on the fracture morphology and bone quality, either a 1/3 tubular plate (DePuy Synthes, Oberdorf, Switzerland), a 1/3 tubular locking compression plate (LCP) (DePuy Synthes, Oberdorf, Switzerland), a Sidewinder Plate System (Trimed, Santa Clarita, California), or a preformed distal fibula LCP (DePuy Synthes, Oberdorf, Switzerland) was used.

In the MIPO group, the correct dimension of the plate (in these cases only LCP 1/3 tubular plate or preformed distal fibula LCP) was chosen based on preoperative radiographic planning. A tourniquet was used for the whole time of operation with a pressure of 100 mmHg above the systolic arterial pressure of the patient. Under fluoroscopic control, the tip of the malleolus was identified, and a slightly curved incision of 2-cm length was made distally to the tip. A distal locking drill sleeve was placed in the plate and used as a grip. Then, the plate was slid subcutaneously along the fibula in a retrograde fashion, care being taken not to create false pathways. Then, a second locking drill sleeve was placed distally and centering the plate onto the fibula with good bone contact; a locking screw was inserted into the most distal plate hole.

**Fig. 1:**
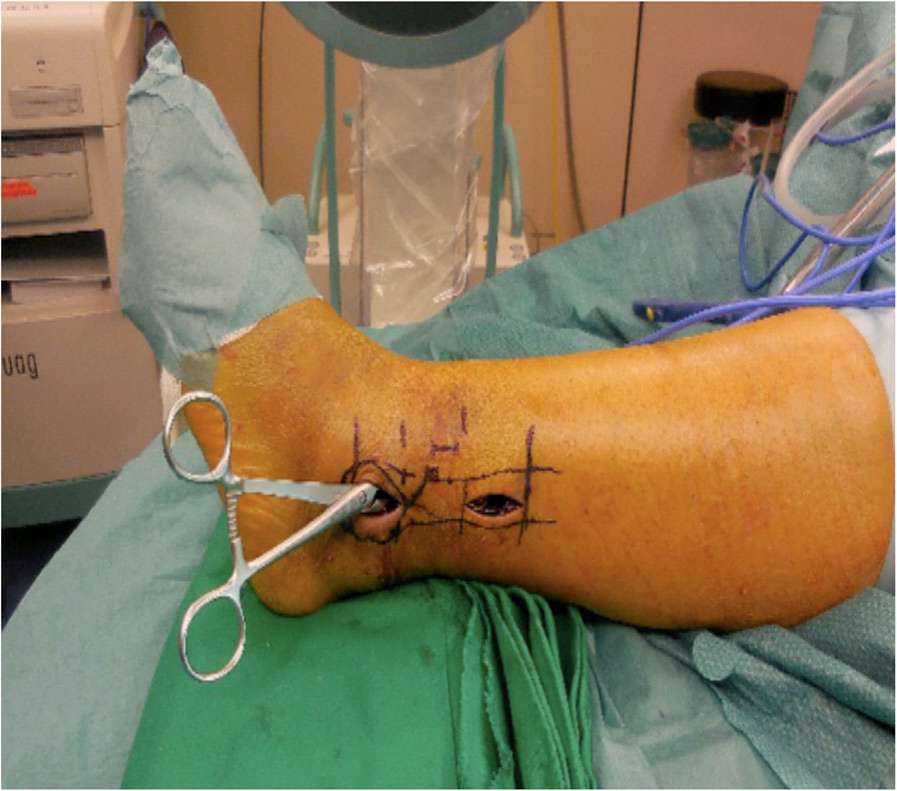
Fracture reduction with assistance of a toothed reduction forceps

**Fig. 2:**
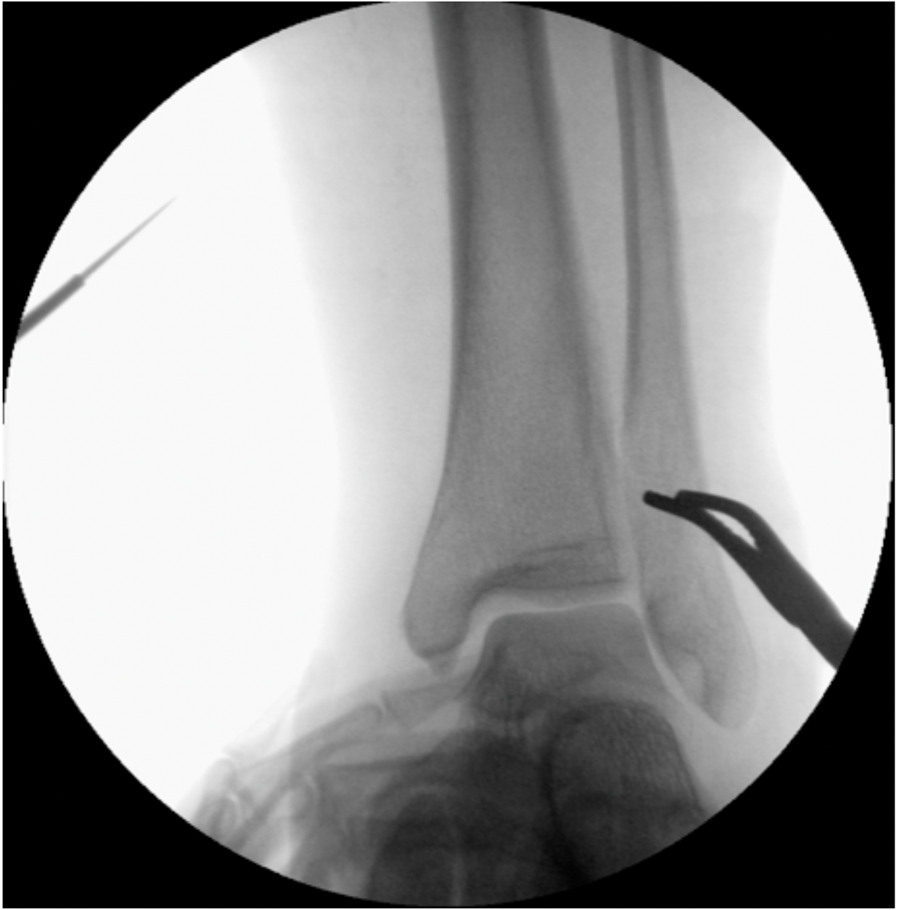
Fluoroscopic control of fibula length and rotation

**Fig. 3:**
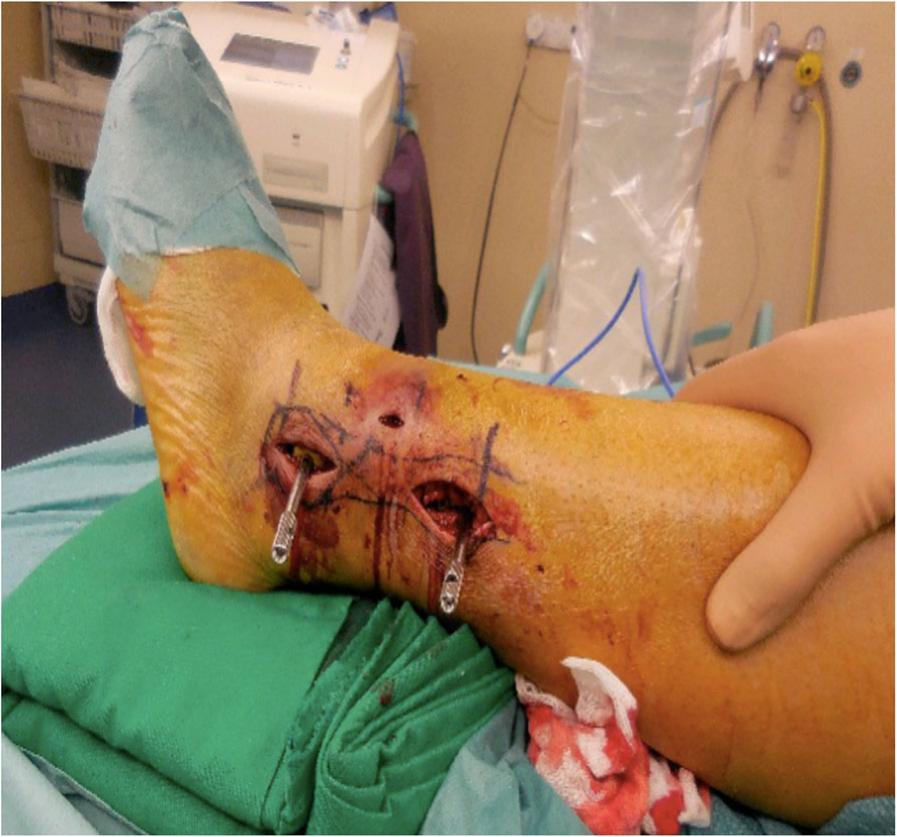
Inserted plate with two drilling sleeves after lag screw placement

In some cases, this maneuver already indirectly reduced the fracture. If not, closed reduction was accomplished with assistance of a toothed reduction forceps (Fig. 1). Correct length and rotation of the fibula in relation to the talus and distal tibia were assessed under fluoroscopic control (Fig. 2). Fractures on frontal plane were reduced with a 2.7-mm bicortical lag screw through a stab incision of the skin and placed perpendicular to the fracture. After reduction, the plate was set with locking head screws through a small 2 cm incision made over the proximal portion of the plate (Fig. 3).

**Fig. 4:**
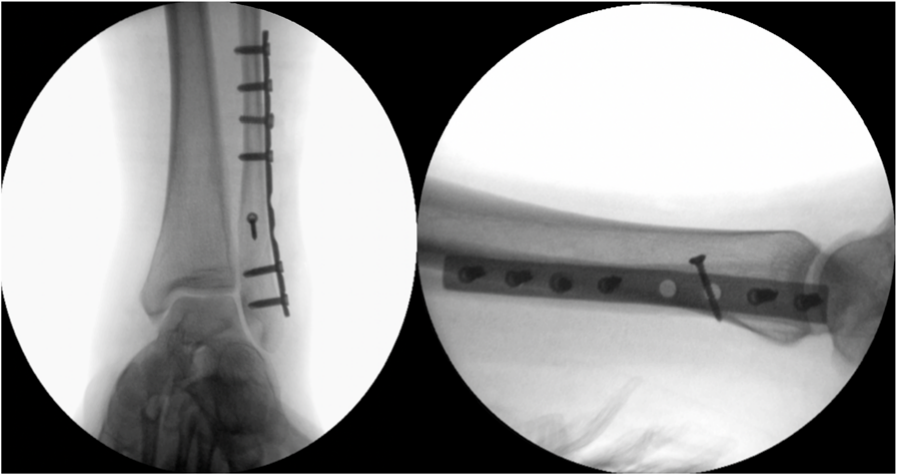
Intraoperative radiologic documentation of plate and screw placement in mortise and lateral view

**Fig. 5:**
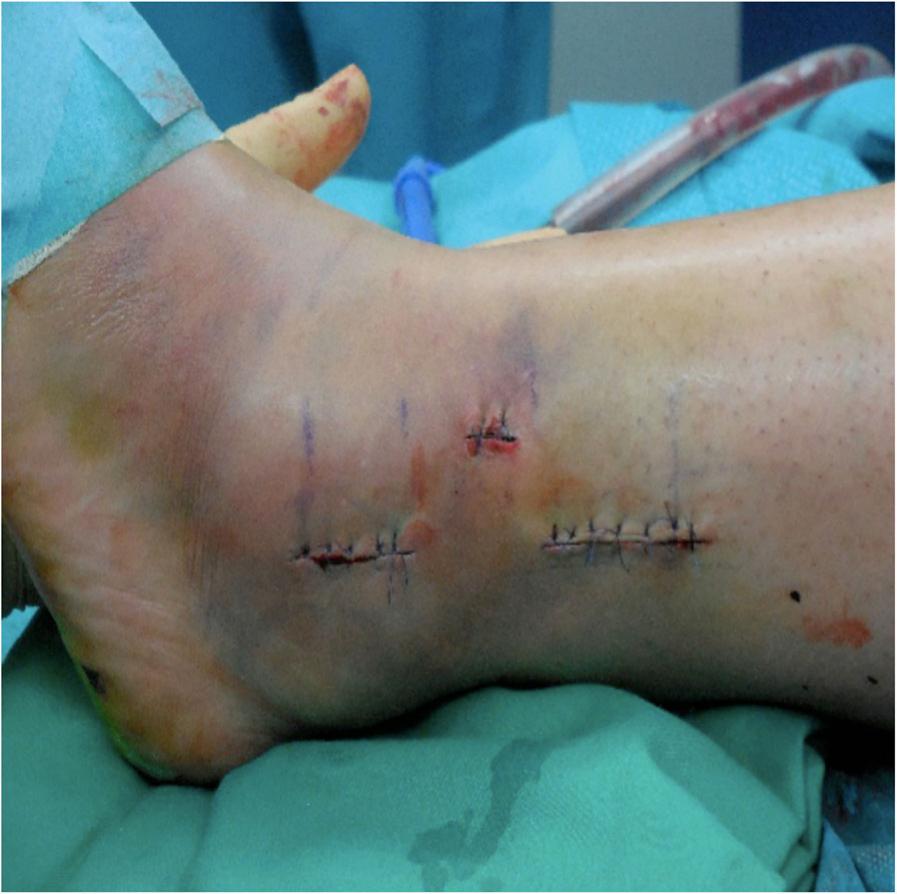
Sutured skin after minimally invasive plate osteosynthesis

Correct position of plate and screws was fluoroscopically documented in mortise and lateral views (Fig. 4). In both groups, a stress testing on the ankle was performed to assess syndesmotic stability. In case of instability of the AITFL and PITFL (anterior and posterior inferior tibiofibular ligament) the syndesmosis was stabilized using two tricortical screws through plate holes. After syndesmotic stabilization, another stress radiograph was performed, and in case medial clear space showed to be above 5 mm, we performed a deltoid ligament repair with a Corkscrew FT Suture Anchor (Arthrex Inc, Naples, USA). Skin closure was performed in a standard manner with non-absorbable sutures (Fig. 5).

### Postoperative management

Both groups and all patients underwent the same postoperative management and follow-up protocol. All ankles were immobilized in a VACOPed walker (Oped, Cham, Switzerland) with the foot at 90° flexion and partial weight bearing was allowed with a load of 15 kg for 6 weeks. Range of motion training was initiated after 2 weeks in case wound healing allowed it. Full weight bearing was allowed 6 weeks postoperatively for radiographically consolidated fractures. In case of trans-syndesmotic fixation, the screws were removed 10 to 12 weeks postoperatively.

### Follow-up

Clinical and radiographic follow-up was done 6 weeks, 3 months, 6 months, and 1 year after surgery. Operation time measured between incision and wound closure as well as length of stay after surgery was noted.

Pain scores were recorded at 1-year follow-up. All patients were assessed using a visual analog scale (VAS) for pain (0–10). VAS was classified into 4 groups: “no pain” for VAS = 0, “low” for VAS = 1–3, “moderate” for VAS = 3–5, and “severe” for VAS = 5–10.

Postoperative complications were recorded and defined as postoperative skin necrosis, nonunion, fracture-related infections, wound healing disorders, and vascular-nerve injuries. Nonunion was defined as a fracture that has not completely healed within 9 months after injury or did not show any signs of healing for 3 consecutive months [23].

Fractures were independently assessed for union in plain radiographs by the operating surgeon as well as by a trained radiologist. Bone healing was defined as absence of pain during weight bearing and bridging of at least three out of four cortices on both the anteroposterior and lateral view. In the event of uncertainty, a computed tomography scan was performed. Any disagreement between the operating surgeon and the radiologist was resolved by consensus.

To assess radiological outcomes, angular and spatial factors, containing the talocrural angle, lateral and medial clear space, tibiofibular overlap, and talar tilt angle were measured in mortise view (anteroposterior view with 15° internal rotation) and compared in both procedures (Table 4).

### Statistical analysis

Data management and statistical analysis were performed using SPSS (IBM Corp. Released 2016. IBM SPSS Statistics for Windows, Version 24.0. Armonk, NY: IBM Corp.). Continuous measures were summarized in terms of mean values and standard deviations, and these values were compared between the two groups using two-tailed unpaired *t* tests. Countable variables were reported as counted values and relevant percentages, and they were compared between groups using Chi-Square test (*χ*^2^).

The alpha level for the statistically significant threshold, for all the tests, was set at 0.05, and when significant, the *p* values are reported in bold in tables. A power analysis was conducted on the percentage of complications in the two groups using an alpha level set at 5%, finding a power of 80% for the performed analysis.

## Results

### Pain assessment

Out of 35 patients, there were nine patients (25.7%) in the ORIF group with postoperative pain at the 1-year follow up (*n* = 2 severe, *n* = 3 moderate, *n* = 4 low pain) versus six out of 35 patients (17.1%) in the MIPO group (*n* = 2 moderate, *n* = 4 low pain). The difference was not statistically significant (*p* = 0.500).

### Complications

Surgery-related complications such as skin necrosis, nonunion, fracture-related infections, wound healing disorders, and vascular-nerve injuries were found more frequently in the ORIF group. However, the difference was not statistically significant. Only the total complication rate including severe pain was significantly lower in the MIPO group compared to the ORIF group, 14% versus 37% (*p* = 0.029) (Table 2).

**Table 2:** Incidence of postoperative pain and complications

Factor	MIPO (***n*** = 35)	ORIF (***n*** = 35)	***t*** test	***p*** value
Postoperative pain (*n*)	6 (17%)	9 (26%)	I^2^ = 2.364	0.500
Severe pain	0 (0%)	2 (6%)		
Moderate pain	2 (6%)	3 (9%)		
Low pain	4 (11%)	4 (11%)		
Soft tissue complications (*n*)	1 (3%)	3 (9%)	*I*^2^ = 1.192	0.275
Nonunion (*n*)	0 (0%)	2 (6%)	*I*^2^ = 2.185	0.139
Superficial and deep wound infection (*n*)	3 (9%)	7 (20%)	*I*^2^ = 2.164	0.141
Vascular-nerve injuries (*n*)	1 (3%)	0 (0%)	*I*^2^ = 0.957	0.328
Total number of patients with complications or severe pain (*n*)	5(14%)	13 (37%)	*I*^2^ = 4.786	**0.029**

All variables were reported in terms of counted cases and relevant percentages and compared with the *I*^2^ test

### Operative time

The mean operative time was significantly longer in the ORIF group—83.2 (± 40.7) minutes versus 66.1 (± 29.5) minutes in the MIPO group (*p* = 0.048). Length of stay was likewise longer in the ORIF group—12.4 (± 8.2) days versus 10.2 (± 4.6) days in the MIPO group, but the difference was not statistically significant (*p* = 0.164) (Table 3).

**Table 3:** Time-related factors

Factor	MIPO	ORIF	***t*** test	***p*** value
Operative time (min)	66.1 ± 29.5	83.2 ± 40.7	*t* = − 2.010	**0.048**
Length of stay (days)	10.2 ± 4.6	12.4 ± 8.2	*t* = − 1.406	0.164

Mean ± standard deviations for the two groups, and relevant comparison performed by means of *t* test

### Radiological outcomes

There were no significant differences between the ORIF and the MIPO group in talocrural angle (77.3° ± 3.1 vs 78° ± 2.4°), lateral clear space (4.9 mm ± 1.2 vs 4.6 mm ± 1.4), medial clear space (3 mm ± 0.8 vs 2.9 mm ± 0.8), and talar tilt angle (0.1° ± 1.1 vs 0.1° ± 1.2). Only the tibiofibular overlap showed a statistically significant difference between the two groups (2.7 mm ± 0.9 vs 3.3 mm ± 1.3, *p* = 0.033) (Table 2).

No case of bone nonunion was observed in the MIPO group compared to two patients in the ORIF group (0% vs. 6%, *p* = 0.139). Nonunion was radiologically defined as absence of bridging of at least three out of four cortices on both the anteroposterior and lateral view (Table 4).

**Table 4:** Postoperative radiologic measures

Factor	MIPO	ORIF	***t*** test	***p*** value
Talocrural angle (°)	77.3 ± 3.1°	78.0 ± 2.4°	*t* = − 1.119	0.267
Lateral clear space (mm)	4.9 ± 1.2	4.6 ± 1.4	*t* = 1.001	0.320
Medial clear space (mm)	3.0 ± 0.8	2.9 ± 0.8	*t* = 0.415	0.679
Tibiofibular overlap (mm)	3.3 ± 1.3	2.7 ± 0.9	*t* = 2.180	**0.033**
Talar tilt angle (°) ^a^	0.1 ± 1.1°	0.1 ± 1.2°	*t* = − 0.211	0.834

Mean ± standard deviations for the two groups and relevant comparison performed by means of *t*-test

^a^ + for varus, − for valgus

## Discussion

The most important finding of the present study was that MIPO led to a lower total complication rate when compared to ORIF in the surgical treatment of distal fibula fractures. Even though not statistically significant, surgery-related complications such as skin necrosis, nonunion, infections, and wound healing disorders as well as vascular-nerve injuries were found more frequently in the ORIF group. Lower postoperative pain scores were noted in the MIPO group. Operative time and length of stay were longer in the ORIF group. Postoperative radiographic measures, except the tibiofibular overlap, showed to be equivalent in both groups.

Since the introduction of minimally invasive plate osteosynthesis (MIPO) following a biological fixation principle by internal bridging of the fracture with preservation of soft tissue, this technique was successfully applied in the treatment of different long bone fractures [3, 4, 13, 14, 24–32].

In open techniques, wound complications remain among the biggest problems to overcome. Currently, reported soft tissue complications range between 17.5 and 22% [4–6, 33]. In elderly patients, complication rates concerning soft tissue might rise as high as 40% [7].

When taking into account concomitant systemic diseases, particularly diabetes and neuropathy, patients have even 3.8 times increased risk of overall complications compared to healthy patients with ankle fractures [34].

MIPO seems to be a reasonable solution to solve the problem of soft tissue complications, and an increasing number of publications about its use in the treatment of long bone fractures seem to underline this trend.

However, only few case series with limited number of patients have been done so far investigating MIPO in distal fibula fractures.

Krenk et al. [3] reported a series of 19 complex ankle injuries treated with MIPO that all healed without skin complications. Historically, many patients complained of pain also due to retained hardware because of the subcutaneous plate placement. No such case was reported in their study, and this may be secondary to the fact that in more than half of the cases a distal screw was not set in the plate.

Hess and Sommer could show the successful application of MIPO in 20 complex distal fibular fractures with critical soft tissue conditions. Of those 20 cases, seventeen fractures healed without complications at all [8].

The only study done so far directly comparing ORIF and MIPO in the treatment of distal fibula fractures was the study done by Iacobellis et al. [4] They could show that none of the MIPO cases had any wound complications in comparison with 5 cases of wound complications in the ORIF group. With only 18 patients in each group, this was nevertheless a rather small cohort study.

The present study consists of 35 complex fibular fractures treated with MIPO and 35 complex fibula fractures treated with ORIF in a single institution. Our rate of complications following the minimally invasive procedure is strongly comparable to the ones above. The rate of total complications in the MIPO group is statistically lower than in the ORIF group (14% vs. 37%, *p* = 0.029). Each complication rate, including infection, skin necrosis, and nonunion showed to be lower in the MIPO group, even though not statistically significant.

In our series, residual postoperative pain was found in 26% of patients in the ORIF group and in 17% of patients in the MIPO group. The cause of postoperative ankle residual pain can be multifactorial. It is frequently related to chondral injuries and soft-tissue impingement as well as posttraumatic neuromas, arthrofibrosis, malreduction, wound infection, and nonunion [35–38]. Our series showed higher rates of nonunion and wound infection in the ORIF group, which could be a contributing factor of higher postoperative pain scores.

Brown et al. were able to show that patients without ankle pain after ORIF of unstable ankle fractures have better functional outcomes than patients who are experiencing significant hardware-related pain [38]. Even though this study did not assess postoperative functional outcomes, it can be assumed that higher postoperative pain rates in the ORIF group would have altered the ankle function to a large extent.

To obtain correct joint congruency, prevent osteoarthritis, and obtain satisfying clinical results after ankle fracture, proper anatomic alignment of the ankle mortise plays a key role [39–42].

In our study, all postoperative radiographic values in both the MIPO and the ORIF group showed to be in the normal, widely accepted range. Nevertheless, those radiographic values have to be interpreted critically not only because there is a substantial variability in normal anatomy between individuals, but also because of recent studies showing that two-dimensional radiographs are not reliable to rule out syndesmotic injury.

Hermans et al. showed that tibiofibular overlap did not correlate with syndesmotic injury, nor did a widened medial clear space correlate with deltoid ligament injury. But whenever measurements deviated, syndesmotic injury was always present, whereas normal measurements did not exclude syndesmotic injury [43]. Those findings are supported by an MRI study showing that a normal tibiofibular radiographic relationship does not preclude syndesmosis disruption and resulting instability [44]. In return, stress radiographs are described to have good reliability [45, 46]. So it was assumed that the majority of syndesmotic instabilities were recognized and addressed with a syndesmotic screw.

The observed statistically significant difference of the tibiofibular overlap between MIPO and ORIF might be accidental, with a large variety of this value described in the literature [47]. In general, the tibiofibular overlap should be greater than or equal to 10 mm [48]. Measurements in both groups can therefore be considered normal.

The closer the postoperative angular and spatial values are to the contralateral healthy ankle the better. Unfortunately, our follow-up protocol did not include a standard radiograph of the healthy side. However, with five different measurements being in the normal range, it could be expected that correct anatomic alignment was achieved in both groups.

Operative time showed to be shorter in the MIPO group compared to ORIF (66.1 vs. 83.2 min, *p* = 0.048). These results are supported by one previous study that also reported shorter operation time in MIPO [4]. When learning a new procedure, performance tends to improve with experience, especially in minimally invasive techniques. The four senior surgeons who performed the operations in this study have several years of experience in MIPO on the distal fibula. Surgeons inexperienced with MIPO, on the early phase of their learning curve, might be faster with ORIF. Shorter operation time in MIPO is only expected with increasing experience.

The main limitation of this study is its retrospective design. Due to its retrospective design and a relative short follow-up period of 1-year, long-term functional outcomes and patient-reported outcome measures were not assessed. It is also limited by a relatively small number of patients, even though it is the largest cohort published so far. The mix of different severity of malleolar fracture types included might be a possible confounding factor. However, different fracture types in our study were represented homogenously in both groups. Finally, lack of comparison of postoperative radiographic measurements with the contralateral side limits the interpretation of those values. To strengthen evidence, prospective randomized and controlled studies with a standardized postoperative evaluation (CT scan or X-ray of the opposite side) as well as standardized collection of specific postoperative functional scores and patient-reported outcome measures are indispensable.

## Conclusion

Our data, experience, and the reviewed medical literature on the topic lead us to the conclusion that MIPO technique for distal fibular fractures should be preferred when condition of soft tissue is critical.

Without doubt, the described advantage of MIPO in comparison with ORIF in the treatment of distal fibula fractures has to be examined and discussed further. With more studies on this topic published in future, evidence will be more meaningful. The results of this study, showing MIPO as a superior technique compared to ORIF in the treatment of distal fibula fractures, can be seen as a trend, which has to be further investigated.

## Data Availability

The datasets generated and/or analyzed during the current study are not publicly available due to respect of individual privacy but are available from the corresponding author on reasonable request.

## Abbreviations

MIPO: Minimally invasive plate osteosynthesis
ORIF: Open reduction and internal fixation
AO: Arbeitsgemeinschaft für Osteosynthesefragen
VAS: Visual analogue scale
LISS: Less invasive stabilization system
LCP: Locking compression plate
OTA: Orthopaedic Trauma Association
AITFL: Anterior inferior tibiofibular ligament
PITFL: Posterior inferior tibiofibular ligament
EKNZ: Ethikkommission Nordwest- und Zentralschweiz

## References

[1] Michelson JD (1995). Fractures about the ankle. J Bone Joint Surg Am.

[2] Siegel J, Tornetta P (2007). Extraperiosteal plating of pronation-abduction ankle fractures. J Bone Joint Surg Am.

[3] Krenk DE, Molinero KG, Mascarenhas L, Muffly MT, Altman GT (2009). Results of minimally invasive distal fibular plate osteosynthesis. J Trauma.

[4] Iacobellis C, Chemello C, Zornetta A, Aldegheri R (2013). Minimally invasive plate osteosynthesis in type B fibular fractures versus open surgery. Musculoskelet Surg.

[5] El-Rayes MA, Hamouda A, Fahmy MAL (1998). Assessment of the results of surgical treatment in displaced ankle fractures. Foot.

[6] Hoiness P, Engebretsen L, Stromsoe K (2003). Soft tissue problems in ankle fractures treated surgically. A prospective study of 154 consecutive closed ankle fractures. Injury..

[7] Wang TJ, Ju WN, Qi BC (2017). Novel management of distal tibial and fibular fractures with Acumed fibular nail and minimally invasive plating osteosynthesis technique: a case report. Medicine (Baltimore).

[8] Hess F, Sommer C (2011). Minimally invasive plate osteosynthesis of the distal fibula with the locking compression plate: first experience of 20 cases. J Orthop Trauma.

[9] Farouk O, Krettek C, Miclau T, Schandelmaier P, Guy P, Tscherne H (1997). Minimally invasive plate osteosynthesis and vascularity: preliminary results of a cadaver injection study. Injury..

[10] Rhinelander FW (1968). The normal microcirculation of diaphyseal cortex and its response to fracture. J Bone Joint Surg Am.

[11] Farouk O, Krettek C, Miclau T, Schandelmaier P, Guy P, Tscherne H (1999). Minimally invasive plate osteosynthesis: does percutaneous plating disrupt femoral blood supply less than the traditional technique?. J Orthop Trauma.

[12] Sommer C, Bereiter H (2005). Actual relevance of minimal invasive surgery in fracture treatment. Ther Umsch.

[13] Krettek C, Schandelmaier P, Miclau T, Bertram R, Holmes W, Tscherne H (1997). Transarticular joint reconstruction and indirect plate osteosynthesis for complex distal supracondylar femoral fractures. Injury..

[14] Krettek C, Schandelmaier P, Miclau T, Tscherne H (1997). Minimally invasive percutaneous plate osteosynthesis (MIPPO) using the DCS in proximal and distal femoral fractures. Injury..

[15] Wenda K, Runkel M, Degreif J, Rudig L (1997). Minimally invasive plate fixation in femoral shaft fractures. Injury..

[16] Gupta P, Tiwari A, Thora A, Gandhi JK, Jog VP (2016). Minimally invasive plate osteosynthesis (MIPO) for proximal and distal fractures of the tibia: a biological approach. Malays Orthop J.

[17] Huang Z, Liu L, Tu C, Zhang H, Fang Y, Yang T (2014). Comparison of three plate system for lateral malleolar fixation. BMC Musculoskelet Disord.

[18] Saxena A, Yun A (2017). Percutaneous plating of Weber B fibular fractures. J Foot Ankle Surg.

[19] von Elm E, Altman DG, Egger M, Pocock SJ, Gøtzsche PC, Vandenbroucke JP (2014). The strengthening the reporting of observational studies in epidemiology (STROBE) statement: guidelines for reporting observational studies. Int J Surg.

[20] Fracture and dislocation compendium (1996). Orthopaedic trauma association committee for coding and classification. J Orthop Trauma.

[21] Marsh JL, Slongo TF, Agel J, Broderick JS, Creevey W, DeCoster TA (2007). Fracture and dislocation classification compendium - 2007: Orthopaedic trauma association classification, database and outcomes committee. J Orthop Trauma.

[22] Buckley RE, Moran CG, Apivatthakakul T, Foundation AO (2017). AO principles of fracture management.

[23] United States Food and Drug Administration (USFDA), Office of Device Evaluation, Guidance document for industry and CDRH staff for the preparation of investigational device exemptions and premarket approval application for bone growth stimulator devices. 1988.

[24] Lau TW, Leung F, Chan CF, Chow SP (2008). Wound complication of minimally invasive plate osteosynthesis in distal tibia fractures. Int Orthop.

[25] Helfet DL, Shonnard PY, Levine D, Borrelli J (1997). Minimally invasive plate osteosynthesis of distal fractures of the tibia. Injury.

[26] Schandelmaier P, Partenheimer A, Koenemann B, Grun OA, Krettek C (2001). Distal femoral fractures and LISS stabilization. Injury.

[27] Collinge C, Protzman R (2010). Outcomes of minimally invasive plate osteosynthesis for metaphyseal distal tibia fractures. J Orthop Trauma.

[28] Gupta RK, Rohilla RK, Sangwan K, Singh V, Walia S (2010). Locking plate fixation in distal metaphyseal tibial fractures: series of 79 patients. Int Orthop.

[29] Redfern DJ, Syed SU, Davies SJ (2004). Fractures of the distal tibia: minimally invasive plate osteosynthesis. Injury..

[30] Zou J, Zhang W, Zhang CQ (2013). Comparison of minimally invasive percutaneous plate osteosynthesis with open reduction and internal fixation for treatment of extra-articular distal tibia fractures. Injury..

[31] Gülabi D, Bekler Hİ, Sağlam F, Taşdemir Z, Çeçen GS, Elmalı N (2016). Surgical treatment of distal tibia fractures: open versus MIPO. Ulus Travma Acil Cerrahi Derg.

[32] Sohn HS, Kim WJ, Shon MS (2015). Comparison between open plating versus minimally invasive plate osteosynthesis for acute displaced clavicular shaft fractures. Injury..

[33] Schepers T, Van Lieshout EM, De Vries MR, Van der Elst M (2011). Increased rates of wound complications with locking plates in distal fibular fractures. Injury..

[34] Wukich DK, Joseph A, Ryan M, Ramirez C, Irrgang JJ (2011). Outcomes of ankle fractures in patients with uncomplicated versus complicated diabetes. Foot Ankle Int.

[35] Dawe EJ, Jukes CP, Ganesan K, Wee A, Gougoulias N (2015). Ankle arthroscopy to manage sequelae after ankle fractures. Knee Surg Sports Traumatol Arthrosc.

[36] Utsugi K, Sakai H, Hiraoka H, Yashiki M, Mogi H (2007). Intra-articular fibrous tissue formation following ankle fracture: the significance of arthroscopic debridement of fibrous tissue. Arthroscopy..

[37] Redfern DJ, Sauvé PS, Sakellariou A (2003). Investigation of incidence of superficial peroneal nerve injury following ankle fracture. Foot Ankle Int..

[38] Brown OL, Dirschl DR, Obremskey WT (2001). Incidence of hardware-related pain and its effect on functional outcomes after open reduction and internal fixation of ankle fractures. J Orthop Trauma.

[39] Harper MC, Hardin G (1988). Posterior malleolar fractures of the ankle associated with external rotation-abduction injuries. Results with and without internal fixation. J Bone Joint Surg Am.

[40] Hughes JL, Weber H, Willenegger H, Kuner EH (1979). Evaluation of ankle fractures: non-operative and operative treatment. Clin Orthop Relat Res.

[41] Pettrone FA, Gail M, Pee D, Fitzpatrick T, Van Herpe LB (1983). Quantitative criteria for prediction of the results after displaced fracture of the ankle. J Bone Joint Surg Am.

[42] Phillips WA, Schwartz HS, Keller CS, Woodward HR, Rudd WS, Spiegel PG (1985). A prospective, randomized study of the management of severe ankle fractures. J Bone Joint Surg Am.

[43] Hermans JJ, Wentink N, Beumer A, Hop WC, Heijboer MP, Moonen AF (2012). Correlation between radiological assessment of acute ankle fractures and syndesmotic injury on MRI. Skelet Radiol.

[44] Nielson JH, Gardner MJ, Peterson MG, Sallis JG, Potter HG, Helfet DL (2005). Radiographic measurements do not predict syndesmotic injury in ankle fractures: an MRI study. Clin Orthop Relat Res.

[45] Sman AD, Hiller CE, Refshauge KM (2013). Diagnostic accuracy of clinical tests for diagnosis of ankle syndesmosis injury: a systematic review. Br J Sports Med.

[46] Egol KA, Amirtharajah M, Tejwani NC, Capla EL, Koval KJ (2004). Ankle stress test for predicting the need for surgical fixation of isolated fibular fractures. J Bone Joint Surg Am.

[47] Sowman B, Radic R, Kuster M, Yates P, Breidiel B, Karamfilef S (2012). Distal tibiofibular radiological overlap: does it always exist?. Bone Joint Res.

[48] Southerland JT. McGlamry’s comprehensive textbook of foot and ankle surgery. 4th ed: Lippincott Williams & Wilkins; 2013.

